# Total thrombus formation analysis in patients with myeloid neoplasia and thrombocytopenia

**DOI:** 10.1007/s00277-025-06679-2

**Published:** 2025-10-15

**Authors:** Katharina Freitag, Georg-Nikolaus Franke, Maria Weise, Carmen Herling, Tristan Klöter, Marco Herling, Annelie Siegemund, Madlen Jentzsch, Sirak Petros, Reinhard Henschler, Klaus H. Metzeler, Christian Pfrepper

**Affiliations:** 1 Division of Hemostaseology, Department of Hematology, Cellular Therapy, Hemostaseology and Infectiology, University of Leipzig Medical Center, Leipzig, Germany; 2 Division of Hematology and Cellular Therapy, Department of Hematology, Cellular Therapy, Hemostaseology and Infectiology, University of Leipzig Medical Center, Leipzig, Germany; 3 Medical ICU, University of Leipzig Medical Center, Leipzig, Germany; 4 Institute of Transfusion Medicine, University of Leipzig Medical Center, Leipzig, Germany

**Keywords:** T-TAS, Myeloid neoplasia, Leukemia, Thrombocytopenia, Platelet function

## Abstract

Patients with hematologic malignancies often require platelet transfusions, but platelet count alone does not sufficiently predict bleeding risk. T-TAS HD-Chip is designed to evaluate the hemostatic function in whole blood samples from thrombocytopenic patients. This study aimed to investigate T-TAS HD-chip in thrombocytopenic patients with myeloid neoplasia. Samples from thrombocytopenic patients with myeloid neoplasia were prospectively collected. The area under the curve (AUC), occlusion start time and occlusion time of T-TAS, platelet count, von Willebrand factor and thrombin generation were measured, and the association of these markers with platelet transfusion, parenteral nutrition and bleeding events was evaluated. A total of 67 samples were collected from 28 patients. No occlusion in T-TAS was detected in samples with platelet counts ≤20 × 10^9^ per liter but 17 of 35 samples with platelet counts > 20 × 10^9^ per liter showed occlusion, *p* < 0.001. In samples from patients with platelet counts > 20 × 10^9^ per liter, 26.3% with parenteral nutrition showed complete occlusion, compared to 75% without parenteral nutrition, *p* = 0.007. Platelet count and T-TAS AUC were significantly higher one hour after platelet transfusion. T-TAS AUC but not platelet count 12–24 h after transfusion was lower in patients requiring next transfusion within < 5 days compared to those with later transfusions. Our data suggest that occlusion in T-TAS HD-Chip in patients with myeloid neoplasia does not occur in patients with platelet counts ≤20 × 10^9^ per liter. Parenteral nutrition seems to interfere with the occlusion of T-TAS HD chip. T-TAS may be able to predict the need for further platelet transfusions.

**Supplementary information:**

The online version contains supplementary material available at 10.1007/s00277-025-06679-2.

## Introduction

Thrombocytopenia is common in patients with hematologic malignancies such as acute myeloid leukemia or other myeloid neoplasia. It may may be caused by the underlying disease itself or a side-effect of treatment [1]. Therefore, many patients with myeloid neoplasia receive platelet transfusions at some point during their treatment. Platelet transfusions are generally safe, but allergic reactions or other adverse events may occasionally occur. Furthermore, platelet transfusion facilitates the development of antibodies that may affect the efficacy of further transfusions [2]. As the production of platelet concentrates depends on voluntary blood donations and transfusion can be associated with adverse reactions, a prudent use of platelet transfusion is necessary.

Indication for platelet transfusion is usually given when the platelet count falls below a certain threshold, which is normally 10 × 10^9^ per liter in non-bleeding patients undergoing chemotherapy or stem cell transplantation [3, 4]. Transfusion is deemed successful when it leads to a rise in platelet count. However, platelet count alone does not sufficiently predict the likeliness of future bleeding events [5, 6]. Two studies have compared different strategies for platelet transfusion, namely platelet transfusion based on platelet count and platelet transfusion following bleeding symptoms [7, 8]. Although the use of platelet concentrates was reduced in both studies when platelet transfusion was only given in bleeding patients, this strategy was associated with a higher risk of bleeding in the cohort reported by Stanworth et al. [7]. In a German multicenter study, no increase in bleeding events was noted in patients after autologous stem cell transplantation, but patients with acute myeloid leukemia had more non-fatal grade 4 bleeding events [8]. Similarly, omitting platelet count guided platelet transfusion in thrombocytopenic hematologic and intensive care patients before placement of a central venous catheter led to an increase in catheter-associated bleeding [9]. To improve decision models on platelet transfusion, the additional analysis of platelet function and their adhesion, aggregation and secretion may provide more information about the hemostatic conditions in vivo. Laboratory tests like light transmission aggregometry, impedance aggregometry or the platelet function analyzer (PFA) are not reliable for samples with a low platelet count. Furthermore, those do not show platelet function under physiological rheologic conditions and only partly capture the platelets’ role in fibrin formation.

The Total Thrombus Formation Analysis System (T-TAS, Zacros, Tokio) is a flow chamber based system to evaluate the hemostatic function in whole blood samples under physiologic blood flow conditions. In T-TAS, the coagulation of blood in a micro-capillary under continuous pressure generates a pressure-time curve, from which occlusion start time (OST), occlusion time (OT), and area under the curve (AUC) are derived. These parameters provide insights into physiological hemostasis. OST relates to the pressure inside the capillary starting to rise above 10 kPa from baseline, which corresponds to the initial platelet adherence. OT is the time for the flow pressure to increase by 60 kPa from baseline, showing a near complete occlusion of the capillary, resembling the thrombus size. AUC represents the thrombogenicity inside the chip, in which a high AUC value represents a quick formation of a stable thrombus, while a lower AUC values show either a slow thrombus formation or the formation of instable thrombi unable to fully occlude the capillary [10, 11]. There are three chips available for T-TAS. The platelet (PL-chip) uses 25 microcapillaries coated with type I collagen and shear rates of 1500/s to represent the primary hemostasis. The atheroma-chip (AR-chip) is a single 80 μm deep channel coated with collagen and tissue thromboplastin resulting in 600/s shear rates are used to reflect the secondary hemostasis. T-TAS PL- and AR-chip successfully predicted bleeding in patients with cardiovascular disease and anti-platelet medication [12]. Like the AR-chip, the hemodilution (HD)-chip is coated with collagen and tissue factor, but has a 50 μm deep flow-chamber, developed for the analysis of samples with low platelet count. In thrombocytopenic patients in an intensive-care-unit, the results of the HD-chip differed significantly between patients with major and minor bleeding and before and after platelet transfusion [13].

This study investigates the use of the T-TAS HD-chip in thrombocytopenic patients with myeloid neoplasia, focusing on its ability to assess bleeding risk and the impact of platelet transfusion.

## Methods

### Study population

Patients hospitalized at the Leipzig University Hospital between December 2022 and July 2023 due to acute myeloid leukemia (AML), acute promyelocytic leukemia (APL), myelodysplastic syndrome (MDS) or myeloproliferative neoplasia (MPN) with a platelet count of 50 × 10^9^ per liter or less were recruited for this study. Exclusion criteria were the use of hormonal contraceptives, nicotine abuse and pregnancy or breastfeeding. In addition, patients using antiplatelet agents within the previous 7 days, non-steroidal anti-inflammatory drugs (NSAID) or metamizole within the previous 24 h, direct oral anticoagulants within the previous 48 h, unfractionated heparin within the last 4 h, prophylactic low molecular weight heparin (LMWH) within the last 12 h or therapeutic LMWH within the last 24 h were excluded from blood sampling.

Two cohorts of patients were included in the study. Group 1 consisted of plasma samples from patients with an indication for platelet transfusion, either due to a platelet count of less than 10 × 10^9^ per liter, due to bleeding or prior to a planned intervention. Blood samples were collected at three time points: before transfusion (t0), one hour (t1), and 12–24 h after transfusion (t2). The time until the next platelet transfusion was recorded.

Group 2 consisted of patients with a platelet count of 50 × 10^9^ per liter or less but no indication for platelet transfusion. Only one blood sample was taken in group 2.

Each patient could be included up to three times in the study, but not more than twice in the same group. Each set of plasma samples collected in the same group from same patient was considered a separate case in this study. Therefore, one patient could be analyzed for a maximum of three cases.

### Patient assessment

After the first sample was taken, the patients were interviewed and examined daily by the same examiner for five days regarding the clinical bleeding symptoms. Results were documented and classified according to the WHO bleeding assessment scale [14] modified and amended by Slichter et al. [15] and Kaufman et al. [3] (supplementary Table 1). In addition, information on age, gender, treatment regimen, timing of platelet transfusions, medication and parenteral nutrition as well as routine laboratory parameters such as platelet count, leukocyte count, hematocrit and lipemia index were obtained from patient records. Samples were deemed lipemic at a lipemia index of 21 or above, as recommended by the central laboratory of the Leipzig University Hospital.

### Laboratory tests

Samples were collected in 0.109 M Sodium citrate tubes (BD Vacutainer, USA) for T-TAS analysis and 3.2% sodium citrate tubes (S-Monovette, Sarstedt, Germany) for further laboratory tests.

Analysis of the whole blood samples was conducted with T-TAS HD-Chip according to the manufacturer’s instructions within 2 h after blood sampling. Read-out parameters for T-TAS were OST, OT and AUC.

Thrombin generation (TGA) was performed using the Calibrated Automated Thrombogram (Diagnostica Stago, France) with commercially available test kits according to the manufacturer’s instructions on a Fluoroskan Ascent (Thermo Labsystems, Finland) at 360/460nm wavelength activated with 5 pM tissue factor.

Frozen leftover samples were used to determine the following hemostatic parameters: von Willebrand factor (VWF) antigen and VWF activity, factor (F) VIII and FXIII, D-dimer and prothrombin fragments on a BCS XP analyzer (all Siemens Healthineers, Germany).

### Statistical analysis

Statistical analysis was performed using IBM SPSS Statistics, Version 29.0.1.1. Correlations were computed using the Spearman correlation coefficient. Differences between groups were assessed depending on their normal distribution using Chi-Square- or Mann-Whitney-U-Test. The change in T-TAS-results in cases with platelet transfusion was assessed using Friedman and Wilcoxon-Test. A p-value of less than 0.05 was deemed significant.

### Ethical considerations

The study was approved by the Ethics Committee of the Medical Faculty at the University of Leipzig (reference 407/21-ek) and conducted in accordance with the Declaration of Helsinki. All patients provided written informed consent prior to the inclusion into the study.

## Results

A total of 32 eligible patients were included in this study. Four patients were excluded due to withdrawal of consent (*n* = 2), hospital discharge (*n* = 1) and administration of metamizole prior to sample acquisition (*n* = 1).

Finally, 28 patients were included in the analysis. Baseline characteristics are shown in Table 1.

**Table 1:** Patients characteristics. AML: acute myeloid leukemia; MDS: myelodysplastic syndrome; MPN: myeloproliferative neoplasia; APL: acute promyelocytic leukemia

Female sex		*n* (%)	9	32.1
Age		Median (range)	59.5	19–77
Diagnosis				
	AML	n (%)	23	82.1
	MDS	n (%)	2	7.1
	MDS/MPN overlap	n (%)	1	3.6
	MPN	n (%)	1	3.6
	APL	n (%)	1	3.6
Therapy				
	Allogeneic stem cell transplantation	n (%)	15	53.6
	Chemotherapy	n (%)	8	28.6
	Immunotherapy	n (%)	1	3.6
	other	n (%)	4	14.3

According to the study protocol, 12 patients were included twice and one patient three times resulting in 42 included cases.

A total of 21 cases were included in group 1. These consisted of 16 cases with data available at t0, t1 and t2, two cases with data at t0, two with data at t1 and one with data at t0 and t2. Group 2 also consisted of 21 cases. In total, 75 plasma samples were collected. One patient developed sepsis four days prior to platelet transfusion, recovered well under antibiotic therapy and was afebrile at the time of platelet transfusion and T-TAS measurement. This patient showed no increase in T-TAS-AUC. Another patient was admitted for treatment of an infection during neutropenia but was afebrile on the day of platelet transfusion. This patient showed an increase in T-TAS-AUC. There were no cases of disseminated intravascular coagulation. Glomerular filtration rate (GFR) was above 50 ml/min in all cases.

### Correlation between T-TAS and platelet count

T-TAS analysis was conducted in 75 samples, platelet count was determined in 67 of those samples. Of those 67 samples, occlusion was observed in 17. None of the 32 samples with a platelet count of 20 × 10^9^ per liter or less showed occlusion. In samples with a platelet count > 20 × 10^9^ per liter, 17 of 35 (48.6%) samples showed occlusion, *p* < 0.001. Therefore, OT was only measurable in 17 and OST in 20 samples.

T-TAS AUC correlated with platelet count when all plasma samples were analyzed (*n* = 67, ρ = 0.507, *p* < 0.001) but did not correlate with platelet count in samples with occlusion (*n* = 17, ρ = 0.216, *p* = 0.405) or with a platelet count > 20 × 10^9^ (*n* = 35, ρ = 0.181, *p* = 0.298). In addition, there was no correlation between OT (*n* = 17, ρ = −0.306 *p* = 0.233) and OST (*n* = 20, ρ = −0.212, *p* = 0.370) with platelet count.

### The influence of parenteral nutrition and lipids on T-TAS

Of 67 samples with known platelet count, 34 were acquired from patients receiving parenteral nutrition rich in lipids. In the 35 samples with platelet counts > 20 × 10^9^ per liter, complete occlusion was seen in 5 (26.3%) samples from patients with parenteral nutrition and in 12 (75.0%) samples from patients without parenteral nutrition, *p* = 0.007.

Samples with a platelet count > 20 × 10^9^ per liter in patients with parenteral nutrition rich in lipids had a median AUC of 44 (*n* = 19, IQR 28–616) compared to 601 (*n* = 16, IQR 203–1073) in patients without parenteral nutrition, *p* = 0.022.

Median OST and OT in patients with and without parenteral nutrition were 862 s (*n* = 6, IQR 459–1202) vs. 1051 s (*n* = 14, IQR 609–1261), *p* = 0.602, and 917 s (*n* = 5, IQR 648–1305) vs. 1154 s (*n* = 12, IQR 821–1415), *p* = 0.279, respectively.

Lipemia was present in 18 of 59 (30.5%) samples, all from patients receiving parenteral nutrition. In samples with platelet count > 20 × 10^9^ per liter, two (25%) samples with lipemia showed occlusion vs. 12 (60%) without lipemia, *p* = 0.209.

Hematocrit did not differ significantly between cases with and without parenteral nutrition (0.210 [IQR 0.199–0.230] vs. 0.222 [IQR 0.205–0.233], *p* = 0.211).

**Fig. 1:**
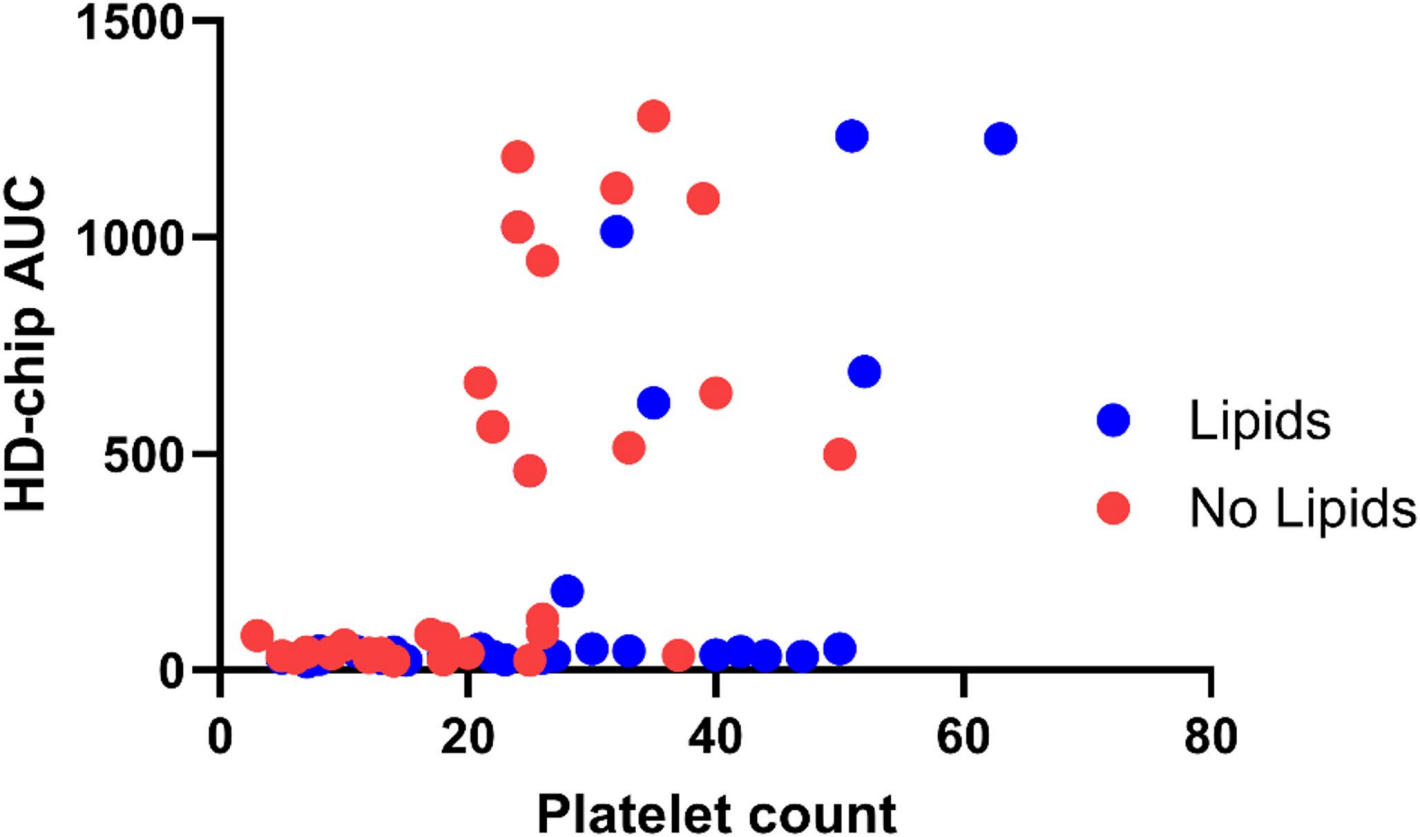
Platelet count (10^9^ per liter) and AUC of the HD-chip in cases with thrombocytopenia, with and without parenteral nutrition rich in lipids

The relation between platelet count and AUC in cases with and without lipids is shown in Fig. 1.

### T-TAS and platelet transfusion

Data before (t0), 1 h (t1) and 12 to 24 h (t2) after platelet transfusion were available in 16 cases. Two cases also received red blood cell concentrate (RBC) transfusion between sample acquisition at t0 and t2. Only one of those cases showed occlusion after platelet and RBC transfusion. There was no documented administration of other blood products, clotting factors or tranexamic acid.

**Fig. 2:**
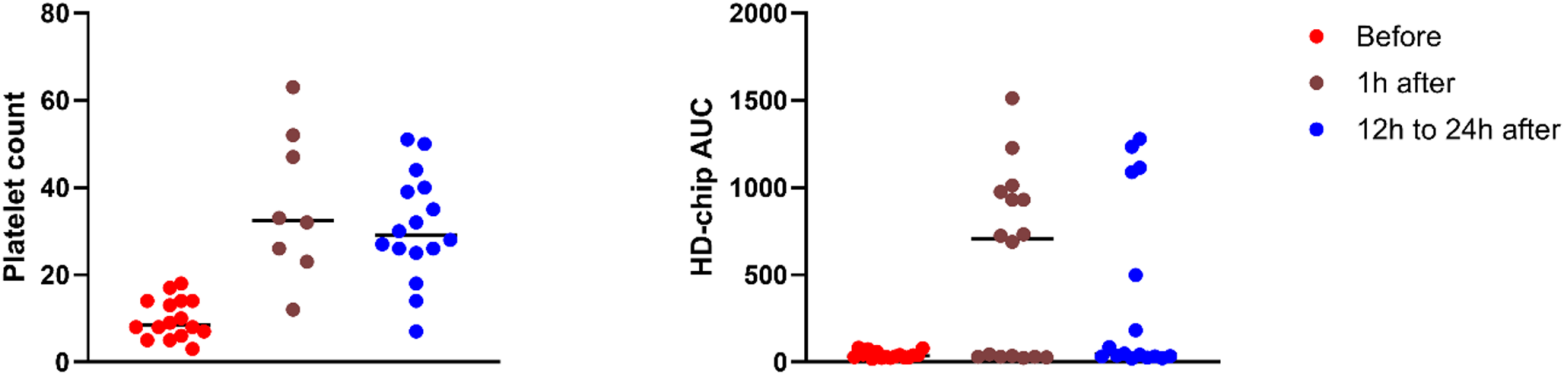
Platelet count (10^9^ per liter) and AUC of the HD-chip before and after platelet transfusion

There was a significant difference in platelet count and T-TAS AUC between t0, t1 and t2. Median (IQR) platelet count was 9 (6–14) x10^9^ per liter at t0, 33 (25–40) x10^9^ per liter at t1 and 29 (25–40) x10^9^ per liter at t2, *p* = 0.002, median (IQR) AUC was 36 (27–55) at t0, 707 (31–964) at t1 and 46 (33–942) at t2, *p* = 0.047, Fig. 2.

Since no occlusion occurred in samples before transfusion, OST and OT were not available at t0. OST and OT 1 h and 12 to 24 h after transfusion did not differ significantly (supplementary Table 2).

**Fig. 3:**
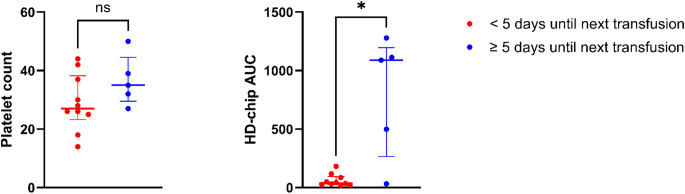
Platelet count (10^9^ per liter) and AUC of the HD-chip 12 h to 24 h after platelet transfusion and time to next transfusion. * *p* < 0.05, ns: not significant

In 19 cases T-TAS and platelet count were measured one day after transfusion. Two cases received no further platelet transfusions due to platelet recovery; in two cases the next transfusion was performed prior to a planned intervention and not due to bleeding or a platelet count < 10 × 10^9^ per liter. From the remaining 15 cases, those receiving the next platelet transfusion within five days (*n* = 10) had a significantly lower AUC at t2 than those with a longer time interval until the next transfusion (38.5 [IQR 25.5–94.0] vs. 1089 [IQR 266–1196], *p* = 0.028), but platelet count did not differ significantly between those groups (27 [IQR 23–38] x 10^9^ per liter vs. 35 [IQR 30–45] x 10^9^ per liter, *p* = 0.156). In the group receiving the next platelet transfusion within five days, 50% had parenteral nutrition rich in lipids, compared to 20% in the other group (*p* = 0.580). The data are summarized in Fig. 3.

### T-TAS and bleeding

In 15 of 42 cases, bleeding classified as WHO grade 1 (*n* = 11) or WHO grade 2 (*n* = 4) was present at the time of blood sampling, bleeding categories are given in the supplementary Table 3. Platelet count differed significantly between samples from cases with WHO grade 2 bleeding compared to those with no bleeding (9 [4–13] x10^9^ per liter vs. 15 [9–24] x10^9^ per liter, *p* = 0.027) but T-TAS AUC was comparable (31.4 [27.9–56.9] vs. 37 [27.1–289}, *p* = 0.755). T-TAS and platelet counts with and without bleeding are shown in the supplementary Table 4. None of the samples from cases with WHO grade 2 bleeding showed occlusion in T-TAS.

### T-TAS and other coagulation parameters

TGA was performed in 59 patient samples without platelet transfusion, as well as before and 12 to 24 h after transfusion. In addition, FVIII, FIX and prothrombin time were determined in 56, VWF antigen, FXII, FXIII, D-Dimers, plasmin-antiplasmin complex (PAP), fibrinogen in 57, activated partial thromboplastin time (aPTT), thrombin time and VWF activity in 47 plasma samples. Due to sample volume restrictions, not all tests could be performed on each sample.

**Fig. 4:**
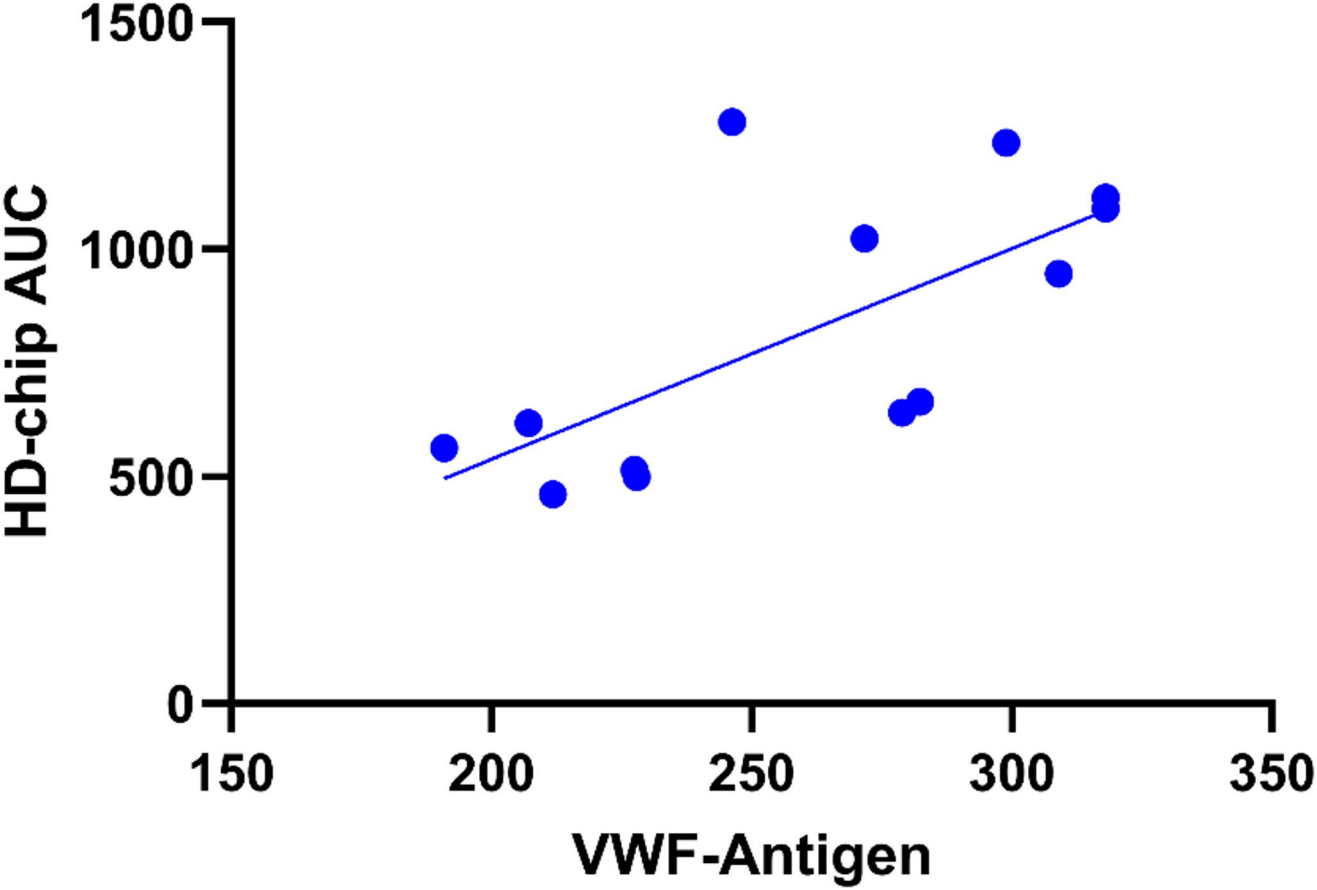
Correlation of the AUC of the HD-chip with von Willebrand factor antigen (IU/dL) in samples with occlusion. VWF: von Willebrand factor

There was a strong correlation of T-TAS parameters with hematocrit, VWF antigen, VWF activity, endogenous thrombin potential (ETP) and D-dimers in samples with occlusion (Table 2; Fig. 4). Other TGA parameters (lag-time, peak thrombin, time to peak, velocity index), FVIII, FIX, FXII, FXIII, plasmin-antiplasmin complex (PAP), fibrinogen, prothrombin time, APTT and thrombin time showed no significant correlation. Detailed results for all measured parameters are shown in the supplementary Tables 5 and 6. The results for patients without parenteral nutrition are given in the supplementary Table 7.

**Table 2:** Correlation of T-TAS in samples with occlusion with hematocrit and coagulation parameters. VWF: von Willebrand factor; ETP: endogenous thrombin potential; * *p* < 0.05 ** *p* < 0.01

		AUC	OST (s)	OT (s)
Hematocrit, *n* = 13	ρ	0.661^*^	−0.714^**^	−0.601^*^
VWF-antigen, *n* = 13	ρ	0.685^**^	−0.652^*^	−0.718^**^
VWF-activity, *n* = 10	ρ	0.661^*^	−0.539	−0.673^*^
ETP, *n* = 14	ρ	−0.499	0.600^*^	0.429
D-dimer, *n* = 14	ρ	0.640^*^	−0.600^*^	−0.701^**^

### Healthy blood donors

Samples from 30 healthy blood donors were analyzed using T-TAS. Results differed significantly between cases without or before platelet transfusion and healthy blood donors. After platelet transfusion, no patient sample reached an AUC higher than the lowest T-TAS AUC measured in a blood donor sample. T-TAS parameters in healthy blood donors compared to samples before or without platelet transfusion are shown in Table 3.

**Table 3:** T-TAS results in samples from healthy blood donors and thrombocytopenic samples. Results are given as median with IQR in brackets

	Healthy blood donors, *n* = 30	Samples before or without platelet transfusion, *n* = 38	*p*-value
AUC	1540 (1486–1566)	40 (28–177)	< 0.001
OST (sec)	243 (217–297)	1085 (738–1177)	< 0.001
OT (sec)	291 (256–348)	1311 (926–1413)	< 0.001

## Discussion

This study aimed to assess the use of T-TAS HD-chip in the prediction of bleeding events and as a potential tool to guide platelet transfusion decisions in thrombocytopenic patients with myeloid neoplasia. We were able to show that the parameters of the T-TAS HD-chip depend on multiple variables, including platelet count, parenteral nutrition, hematocrit and VWF.

In our cohort, occlusion of the HD-chip only occurred in samples with a platelet count of 20 × 10^9^ per liter or more. In platelet counts higher than that, platelet count did not correlate with T-TAS parameters. In experimentally induced thrombocytopenia, Samanbar et al. report statistically significant differences of AUC in groups with high (37–62 × 10^9^ per liter), middle (16–36 × 10^9^ per liter) and low (2–14 × 10^9^ per liter) platelet counts. Even in the low platelet count group, mean AUC was still 1,084.4 ± 72.5 and OT 12.6 ± 1.4 min. In addition, OT was 27.2 ± 1.0 min in pre-transfusion samples of patients with hematologic malignancies, mainly AML, with an average platelet count of 10.8 ± 0.6 × 10^9^ per liter [16]. Therefore, Samanbar et al. reported occlusion with lower platelet counts than in our cohort, suggesting that external factors, such as parenteral nutrition, may influence T-TAS results considerably.

Parenteral nutrition is common in hematology patients if enteral nutrition is impossible due to mucositis [17]. Samples from patients receiving parenteral nutrition rich in lipids showed significantly less occlusion than other samples, possibly due to interference of the lipids with T-TAS chip or direct effects on coagulation in vivo. Lipemia is known to affect a variety of coagulation tests [18] and lipid emulsions have been shown to influence platelet function [19] and bleeding tendency in vivo [20]. In addition, parenteral nutrition may alter T-TAS measurements, leading to a misinterpretation of platelet function. Possible mechanisms include an increase in sedimentation, leading to a change of viscoelastic properties and contact time of platelets with collagen and tissue factor in the chip and therefore altered platelet adhesion and aggregation. Despite the use of 4,4’-Dinitrostilbene-2,2’-disulfonic acid disodium salt (DNDS) as an anti-sedimentation agent, visible sedimentation occurred in several samples in this study.

Samanbar and colleagues documented measurable occlusion in their cohort of patients with mainly hematologic diseases (52% with myeloid neoplasms) undergoing chemotherapy and stem cell transplantation. Unfortunately, it is not documented whether those patients received parenteral nutrition, which limits the comparability of both studies [16].

Atari et al. reported an AUC of < 100 before and after platelet transfusion in one sample from a patient with AML. This was the only patient with a hematologic malignancy in that cohort and the only one without recovery of T-TAS AUC after platelet transfusion. Unfortunately, OST, OT and the use of parenteral nutrition were not reported, so it can only be speculated whether the lack of increase in AUC was caused by parenteral nutrition or had another reason [13].

Besides platelet count and parenteral nutrition, hematocrit and VWF also appear to have a significant influence on T-TAS results. Samanbar and colleagues showed differences in T-TAS AUC between samples with a hematocrit above or below 0.25 [16]. In our study, hematocrit correlated with AUC as well as inversely with OT and OST in samples with occlusion, supporting these findings. Decreased hematocrit is associated with an increased bleeding risk in thrombocytopenic patients undergoing chemotherapy or stem cell transplantation [6]. Red blood cell transfusion alone may improve coagulation in anemic patients [21]. Other tests, such as the Platelet Function Analyzer (PFA) are influenced by the hematocrit and may only be used above certain hematocrit thresholds [22]. Since T-TAS results before and after red blood cell transfusions were only available from two patients, we cannot draw any conclusion from our results.

We found that an increase in VWF-concentration and activity correlates with faster occlusion in the T-TAS HD-chip. In patients with liver disease, an upregulation of VWF is associated with a re-compensation of hemostatic potential despite thrombocytopenia [23]. Endothelial damage and inflammation induce upregulation of VWF in patients with hematologic malignancies as well [24]. VWF showed a stronger correlation with T-TAS than platelet count in patients with a platelet count > 20 × 10^9^ per liter in our study. This suggests that T-TAS reflects the interaction of platelets with VWF and that the effect of thrombocytopenia in the HD-chip may be overcome by the high VWF-activity in our group of patients with myeloid malignancies. This is consistent with the correlation of T-TAS-AUC and D-dimer, which shows that T-TAS may represent the activation of the coagulation cascade.

Previous results of the T-TAS HD chip have shown that it can detect differences between patients with major and minor bleeding and before and after platelet transfusion [13, 16]. While bleeding was not correlated with the results of T-TAS in our study, T-TAS AUC as well as platelet count increased significantly after platelet transfusion. Since occlusion did not occur in samples before platelet transfusion, differences in OST and OT could not be analyzed. Samples from patients who required another platelet transfusion within five days or less had significantly lower T-TAS AUC 12 to 24 h after transfusion compared to those from patients who did not require another transfusion in this period. This difference was not seen for platelet count, suggesting that the T-TAS results 12 to 24 h after platelet transfusion may better represent transfusion success than an increase in platelet count. Of note, 50% of the patients who required another platelet transfusion within five days or less were also more likely to receive parenteral nutrition, which may have influenced these results.

Patients with thrombocytopenia had a significantly lower AUC and longer OT and OST than healthy blood donors. This shows that the T-TAS HD chip can successfully discriminate between thrombocytopenic patients and healthy subjects.

This study has several limitations. The aim to assess, whether T-TAS correlates with bleeding tendency, was not achieved. This is likely due to the relatively small patient cohort and a low number of moderate and severe bleeding episodes. Several laboratory parameters, including platelet counts 1 h after transfusion and VWF parameters were not available from all patients. A larger cohort of patients and availability of parameters from all patients could lead to more robust results.

## Summary

The T-TAS HD chip is able to show the effects of platelet transfusions in patients with myeloid malignancies and may be able to distinguish patients who require further platelet transfusion earlier than others. In addition, T-TAS parameters are correlated with VWF and hematocrit. Parenteral nutrition, which is commonly used in hematologic patients, appears to have a significant impact on T-TAS results. However, our study could not show a correlation between T-TAS results and bleeding risk in our cohort of hospitalized patients. Further research especially in stable outpatients is needed before T-TAS can be considered as a decision-making tool for platelet transfusion in patients with hematologic malignancies.

## Supplementary information

Below is the link to the electronic supplementary material.

Supplementary File 1 (DOCX 43.9 KB)

## Data Availability

The datasets generated and analyzed during the current study are available from the corresponding author on reasonable request.
