# Supplementary material for: Total thrombus formation analysis in patients with myeloid neoplasia and thrombocytopenia
Source: Ann Hematol. 2025 Oct 15;104(10):5453–61. doi: 10.1007/s00277-025-06679-2 (PMC12619771; doi:10.1007/s00277-025-06679-2)
Supplement: Supplementary file 1 — (DOCX 43.9 KB) [file 277_2025_6679_MOESM1_ESM.docx]

Supplementary table 1: WHO Bleeding Score

|  | Grade 1 | Grade 2 | Grade 3 | Grade 4 |
| --- | --- | --- | --- | --- |
| Oral. nasal | Bleeding shorter than 30 min in 24 h | Bleeding longer than 30 min in 24 h  Symptomatic oral blood blisters | Bleeding requiring transfusion within 24 hours due to bleeding | Bleeding requiring transfusion within 24 hours and need for intensive care treatment due to the bleeding  fatal bleeding |
| Skin. soft tissue. musculoskeletal | Local petechia  purpura sized below 2.5 cm  spontaneous hematoma sized above 2.5 cm | Generalized petechia  purpura sized above 2.5cm  Joint bleeding (diagnosed by imaging or aspiration of synovial fluid)  Other tissue bleeding |  |  |
| Gastrointestinal | Occult blood in stool | Melena, Hematemesis, Hematochezia  Stool with blood deposits |  |  |
| Urogenital | Microscopic hematuria/hemoglobinuria  Abnormal vaginal bleeding (spotting) or heavier-than-expected menstrual bleeding | Macroscopic hematuria  Unexpected vaginal bleeding more severe than spotting |  |  |
| Pulmonary |  | Hemoptysis  Blood in BAL, bloody tinged sputum |  |  |
| Wounds |  | Bleeding from the surgical site >1h Duration within 24h  Bleeding from venous puncture site >1h |  |  |
| Retinal |  | Retinal hemorrhage without visual impairment |  | Retinal hemorrhage with visual impairment |
| CNS |  | >5 RBC/µl in CSF, without symptoms, without visible redness of the aspirate | Lumbar puncture with visible redness of the aspirate | Cerebral hemorrhage in imaging with/without symptoms |
| Other |  | Bleeding into the pleura, peritoneal cavity, pericardium without need for transfusion | Bleeding with need for transfusion | Bleeding with need for transfusion and hemodynamic instability |

Supplementary table 2: T-TAS parameters and platelet count before and after platelet transfusion

|  | Before transfusion (t0). Median (IQR) | 1 hour after transfusion (t1). Median (IQR) | 12-24 hours after transfusion (t2). Median (IQR) | p-value |
| --- | --- | --- | --- | --- |
| Platelet count  (x 10^9^ per liter) | 9 (6 - 14)  n=16 | 33 (25 - 40)  n=8 | 29 (25 – 40)  n=16 | p = 0.002  t1-t2: p = 0.008  t1-t3: p = 0.008  t2-t3: p = 1.0 |
| T-TAS AUC | 36 (27 – 55)  n=16 | 707 (31 – 964)  n=16 | 46 (33 – 942)  n=16 | p = 0.047  t1-t2: p = 0.065  t1-t3: p = 0.155  t2-t3: p = 1.0 |
| T-TAS OST (sec) | - | 691 (525 – 1000)  n=9 | 631 (459 - 1551)  n=7 | p = 0.578 |
| T-TAS OT (sec) | - | 1084 (778 - 1207)  n=9 | 813 (679 – 1151)  n=5 | p = 0.625 |

Supplementary table 3: Bleeding at time of inclusion

| WHO bleedings score at time of inclusion | |  |  |
| --- | --- | --- | --- |
|  | 0 | n (%) | 27 (60.5) |
|  | 1 | n (%) | 11 (29.0) |
|  | 2 | n (%) | 4 (10.5) |
| Bleeding category | |  |  |
|  | oral and nasal | n | 11 |
|  | skin | n | 10 |
|  | urogenital | n | 2 |
|  | gastrointestinal | n | 2 |
|  | post-interventional | n | 2 |

Supplementary table 4: T-TAS and platelet count in patients with and without bleeding

|  | Cases without bleeding (median. IQR) | Cases with bleeding (median. IQR) | p-value |
| --- | --- | --- | --- |
| Platelet count  (x 10^9^ per liter) | 15 (9 - 24)  n=27 | 13 (11 - 20)  n=15 | p = 0.439 |
| T-TAS AUC | 37 (27 – 460)  n=27 | 42 (35 - 79).  n=15 | p = 0.409 |
| T-TAS OT (s) | 1300 (845 – 1407)  n=8 | 1381 (1344 – n.a.)  n=2 | p = 0.333 |
| T-TAS OST (s) | 1051 (734 – 1300)  n=9 | 1099 (1085 – n.a.)  n=2 | p = 0.889 |

Supplementary table 5: Correlation between T-TAS parameters and coagulation parameters in samples with occlusion. * p < 0.05 ** p < 0.01; VWF: von Willebrand factor; ETP: endogenous thrombin potential; PAP: Plasmin-antiplasmin complex

|  |  | AUC | OST (s) | OT (s) |
| --- | --- | --- | --- | --- |
| Lag-time (min) | ρ | 0.454 | -0.489 | -0.471 |
|  | p | 0.103 | 0.076 | 0.089 |
|  | n | 14 | 14 | 14 |
| ETP (nM*min) | Ρ | -0.499 | .0600^*^ | 0.429 |
|  | P | 0.069 | 0.023 | 0.126 |
|  | n | 14 | 14 | 14 |
| Peak (nM) | ρ | -0.363 | 0.486 | 0.292 |
|  | p | 0.203 | 0.078 | 0.311 |
|  | n | 14 | 14 | 14 |
| Time to Peak (min) | ρ | 0.409 | -0.473 | -0.412 |
|  | p | 0.146 | 0.087 | 0.144 |
|  | n | 14 | 14 | 14 |
| Velocity Index (nM*min) | ρ | -0.253 | 0.385 | 0.178 |
|  | p | 0.383 | 0.175 | 0.543 |
|  | n | 14 | 14 | 14 |
| PAP | ρ | 0.182 | -0.095 | -0.253 |
|  | p | 0.533 | 0.748 | 0.383 |
|  | n | 14 | 14 | 14 |
| FXII | ρ | 0.160 | -0.165 | -0.240 |
|  | p | 0.584 | 0.573 | 0.409 |
|  | n | 14 | 14 | 14 |
| FIX (%) | ρ | 0.357 | -0.368 | -0.452 |
|  | p | 0.254 | 0.240 | 0.140 |
|  | n | 12 | 12 | 12 |
| D-dimer (mg/l) | ρ | 0.640^*^ | -0.600^*^ | -0.701^**^ |
|  | p | 0.014 | 0.023 | 0.005 |
|  | n | 14 | 14 | 14 |
| FVIII (%) | ρ | 0.095 | -0.051 | -0.156 |
|  | p | 0.748 | 0.864 | 0.594 |
|  | n | 14 | 14 | 14 |
| INR | ρ | 0.383 | -0.425 | -0.299 |
|  | p | 0.219 | 0.168 | 0.346 |
|  | n | 12 | 12 | 12 |
| FXII (%) | ρ | -0.387 | 0.369 | 0.320 |
|  | p | 0.172 | 0.194 | 0.265 |
|  | n | 14 | 14 | 14 |
| VWF-antigen (%) | ρ | 0.685^**^ | -0.652^*^ | -0.718^**^ |
|  | p | 0.010 | 0.016 | 0.006 |
|  | n | 13 | 13 | 13 |
| aPTT (sec) | ρ | -0.029 | 0.011 | 0.020 |
|  | p | 0.923 | 0.970 | 0.946 |
|  | n | 14 | 14 | 14 |
| Fibrinogen (g/l) | ρ | -0.363 | 0.442 | 0.279 |
|  | p | 0.203 | 0.114 | 0.334 |
|  | n | 14 | 14 | 14 |
| VWF-activity (%) | ρ | 0.661^*^ | -0.539 | -0.673^*^ |
|  | p | 0.038 | 0.108 | 0.033 |
|  | n | 10 | 10 | 10 |
| Thrombin time (sec) | ρ | 0.274 | -0.432 | -0.255 |
|  | p | 0.444 | 0.213 | 0.476 |
|  | n | 10 | 10 | 10 |
| Hematocrit | ρ | 0.661^*^ | -0.714^**^ | -0.601^*^ |
|  | p | 0.014 | 0.006 | 0.030 |
|  | n | 13 | 13 | 13 |

Supplementary table 6: Correlation of T-TAS and other hemostatic parameters in all analyzed samples. * p < 0.05 ** p < 0.01; VWF: von Willebrand factor; ETP: endogenous thrombin potential; PAP: Plasmin-antiplasmin complex

|  | | T-TAS AUC | T-TAS OST | T-TAS OT |
| --- | --- | --- | --- | --- |
| Lag-time (min) | ρ | -0.155 | -0.656^**^ | -0.471 |
|  | p | 0.240 | 0.004 | 0.089 |
|  | n | 59 | 17 | 14 |
| ETP (nM*min) | ρ | 0.016 | 0.456 | 0.429 |
|  | p | 0.907 | 0.066 | 0.126 |
|  | n | 59 | 17 | 14 |
| Peak (nM) | ρ | 0.017 | 0.277 | 0.292 |
|  | p | 0.899 | 0.282 | 0.311 |
|  | n | 59 | 17 | 14 |
| Time to Peak (min) | ρ | -0.107 | -0.498^*^ | -0.412 |
|  | p | 0.419 | 0.042 | 0.144 |
|  | n | 59 | 17 | 14 |
| Velocity Index (nM*min) | ρ | 0.011 | 0.181 | 0.178 |
|  | p | 0.935 | 0.486 | 0.543 |
|  | n | 59 | 17 | 14 |
| PAP | ρ | -0.229 | -0.162 | -0.253 |
|  | p | 0.081 | 0.535 | 0.383 |
|  | n | 59 | 17 | 14 |
| FXII | ρ | -0.026 | -0.301 | -0.240 |
|  | p | 0.848 | 0.240 | 0.409 |
|  | n | 59 | 17 | 14 |
| FIX (%) | ρ | 0.252 | -0.633^*^ | -0.452 |
|  | p | 0.061 | 0.011 | 0.140 |
|  | n | 56 | 15 | 12 |
| D-dimer (mg/l) | ρ | -0.299^*^ | -0.525^*^ | -0.701^**^ |
|  | p | 0.022 | 0.031 | 0.005 |
|  | n | 59 | 17 | 14 |
| FVIII (%) | ρ | 0.028 | -0.402 | -0.156 |
|  | p | 0.834 | 0.110 | 0.594 |
|  | n | 59 | 17 | 14 |
| INR | ρ | 0.217 | -0.188 | -0.299 |
|  | p | 0.122 | 0.502 | 0.346 |
|  | n | 52 | 15 | 12 |
| FXII (%) | ρ | 0.002 | 0.263 | 0.320 |
|  | p | 0.989 | 0.308 | 0.265 |
|  | n | 59 | 17 | 14 |
| VWF-antigen (%) | ρ | 0.162 | -0.673^**^ | -0.718^**^ |
|  | p | 0.229 | 0.004 | 0.006 |
|  | n | 57 | 16 | 13 |
| aPTT (sec) | ρ | -.346^**^ | 0.092 | 0.020 |
|  | p | 0.007 | 0.726 | 0.946 |
|  | n | 59 | 17 | 14 |
| Fibrinogen (g/l) | ρ | -0.352^**^ | -0.025 | 0.279 |
|  | p | 0.007 | 0.926 | 0.334 |
|  | n | 57 | 17 | 14 |
| VWF-activity (%) | ρ | 0.210 | -0.469 | -0.673^*^ |
|  | p | 0.157 | 0.124 | 0.033 |
|  | n | 47 | 12 | 10 |
| TZ (sec) | ρ | 0.280 | -0.308 | -0.255 |
|  | p | 0.057 | 0.330 | 0.476 |
|  | n | 47 | 12 | 10 |
| Hematocrit | ρ | 0.191 | -0.827 | -0.601 |
|  | p | 0.151 | < 0.001 | 0.030 |
|  | n | 58 | 16 | 13 |

Supplementary table 7: Correlation of T-TAS and other hemostatic parameters in samples without parenteral nutrition. * p < 0.05 ** p < 0.01; VWF: von Willebrand factor; ETP: endogenous thrombin potential; PAP: Plasmin-antiplasmin complex

|  | | T-TAS AUC | T-TAS OST | T-TAS OT |
| --- | --- | --- | --- | --- |
| Lag-time (min) | ρ | 0.050 | -0.535^*^ | -0.451 |
|  | p | 0.783 | 0.049 | 0.141 |
|  | n | 33 | 14 | 12 |
| ETP (nM*min) | ρ | -0.235 | 0.556^*^ | 0.660^*^ |
|  | p | 0.188 | 0.039 | 0.020 |
|  | n | 33 | 14 | 12 |
| Peak (nM) | ρ | -0.090 | 0.350 | 0.545 |
|  | p | 0.620 | 0.220 | 0.067 |
|  | n | 33 | 14 | 12 |
| Time to Peak (min) | ρ | 0.032 | -0.446 | -0.454 |
|  | p | 0.858 | 0.110 | 0.138 |
|  | n | 33 | 14 | 12 |
| Velocity Index (nM*min) | ρ | -0.030 | 0.285 | 0.537 |
|  | p | 0.870 | 0.324 | 0.072 |
|  | n | 33 | 14 | 12 |
| PAP | ρ | -0.025 | 0.302 | -0.138 |
|  | p | 0.891 | 0.294 | 0.670 |
|  | n | 33 | 14 | 12 |
| FXII | ρ | 0.098 | -0.105 | 0.076 |
|  | p | 0.588 | 0.722 | 0.815 |
|  | n | 33 | 14 | 12 |
| FIX (%) | ρ | 0.251 | -0.094 | -0.348 |
|  | p | 0.181 | 0.750 | 0.325 |
|  | n | 30 | 14 | 10 |
| D-dimer (mg/l) | ρ | -0.058 | -0.649^*^ | -0.518 |
|  | p | 0.750 | 0.022 | 0.085 |
|  | n | 33 | 12 | 12 |
| FVIII (%) | ρ | 0.191 | -0.581^*^ | -0.015 |
|  | p | 0.288 | 0.029 | 0.964 |
|  | n | 33 | 14 | 12 |
| INR | ρ | 0.296 | -0.152 | -0.265 |
|  | p | 0.112 | 0.619 | 0.430 |
|  | n | 30 | 13 | 11 |
| FXII (%) | ρ | -0.395^*^ | 0.663^**^ | 0.598^*^ |
|  | p | 0.023 | 0.010 | 0.040 |
|  | n | 33 | 14 | 12 |
| VWF-antigen (%) | ρ | 0.399^*^ | -0.647^*^ | -0.658^*^ |
|  | p | 0.026 | 0.017 | 0.028 |
|  | n | 31 | 13 | 11 |
| aPTT (sec) | ρ | -0.141 | -0.024 | 0.057 |
|  | p | 0.435 | 0.936 | 0.861 |
|  | n | 33 | 14 | 12 |
| Fibrinogen (g/l) | ρ | -0.341 | 0.207 | 0.600^*^ |
|  | p | 0.056 | 0.477 | 0.039 |
|  | n | 32 | 14 | 12 |
| VWF-activity (%) | ρ | 0.521^*^ | -0.283 | -0.570 |
|  | p | 0.015 | 0.460 | 0.140 |
|  | n | 21 | 9 | 8 |
| Hematocrit | ρ | 0.424^*^ | -0.723^**^ | -0.578 |
|  | p | 0.015 | 0.005 | 0.062 |
|  | n | 32 | 13 | 11 |
